# Ventilator Weaning Failure Induced by Oral Beraprost in a Patient after Femoral Endarterectomy: A Case Report

**DOI:** 10.3400/avd.cr.25-00140

**Published:** 2026-06-02

**Authors:** Masato Obayashi, Shoji Fukuda, Akinari Iwahori, Toru Iwahashi, Toshiki Fujiyoshi, Shun Suzuki, Masaki Kano, Ikki Kojima, Makoto Matsuda, Shinobu Akiyama, Yusuke Shimahara

**Affiliations:** 1 Department of Nurse Practitioner Management Room, Tokyo Medical University Hospital, Tokyo, Japan; 2 Department of Cardiovascular Surgery, Tokyo Medical University Hospital, Tokyo, Japan

**Keywords:** beraprost sodium, ventilator weaning, ventilation–perfusion mismatch

## Abstract

Beraprost sodium, an oral prostacyclin analog, is a systemic vasodilator whose role in ventilator weaning failure has not been widely described. We present the case of a 90-year-old man with extensive atelectasis who received beraprost for critical limb ischemia and developed refractory ventilator weaning failure. Each weaning attempt was interrupted by recurrent episodes of tachypnea and marked hypoxemia. These events resolved after discontinuation of beraprost; however, reintroduction of the drug reproduced identical respiratory deterioration, suggesting a potential association. Oral beraprost may contribute to recurrent hypoxemia and tachypnea in patients with severe pre-existing ventilation–perfusion mismatch, potentially leading to ventilator weaning failure.

## Introduction

Beraprost sodium is an orally available prostacyclin analog with established vasodilatory and antiplatelet effects, primarily indicated for pulmonary arterial hypertension and peripheral arterial disease.^1,2)^ Its therapeutic action is mediated by the prostacyclin receptor, leading to increased cyclic adenosine monophosphate and subsequent smooth muscle relaxation.

However, the nonselective nature of systemic vasodilation can pose significant risks in certain clinical settings. Pulmonary circulation relies on hypoxic pulmonary vasoconstriction (HPV), a critical autoregulatory mechanism that diverts blood flow away from poorly ventilated lung regions, thereby optimizing the ventilation–perfusion (V/Q) ratio and maintaining arterial oxygenation.^3)^ This reflex is essential in patients with marked V/Q mismatch, such as those with atelectasis. Systemic vasodilators can indiscriminately blunt HPV, increasing intrapulmonary shunting and precipitating severe hypoxemia, a well-recognized concern in critical patients.^4)^

Despite these known class effects, the potential of oral beraprost to cause severe respiratory compromise that delays or prevents ventilator weaning has not been described. Pivotal trials often exclude patients with significant pulmonary comorbidities, leaving a gap in safety data for this high-risk population.^2)^ Here, we report a case in which oral beraprost was associated with severe ventilator weaning failure, a finding that highlights a potential patient–drug interaction.

## Case Report

**Fig. 1:**
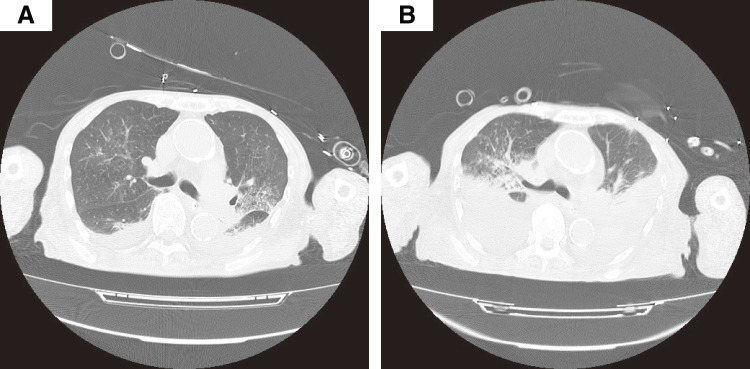
(**A**) Thoracic CT scan on hospital day 7 at the onset of respiratory failure, demonstrating bilateral ground-glass opacities and moderate pleural effusions. (**B**) Follow-up CT scan on hospital day 29 demonstrating a marked interval increase in pleural effusions, resulting in extensive compressive atelectasis. CT: computed tomography

A 90-year-old man (height, 166.5 cm; weight, 41.9 kg; body mass index, 15.1 kg/m^2^) with a history of hypertension and polymyalgia rheumatica was admitted for surgical revascularization of critical limb ischemia. His preoperative pulmonary function test results were generally within normal limits (forced expiratory volume, 87.8%; vital capacity, 75.6%). Preoperative transthoracic echocardiography revealed a left ventricular ejection fraction of 68%, with no wall motion asynergy. Although moderate aortic regurgitation was noted, no other valvular disease was observed. Preoperative chest computed tomography (CT) showed emphysematous changes, predominantly in the bilateral upper lobes, without other significant findings. The patient’s medications included prednisolone 5 mg daily, clopidogrel 25 mg daily, and beraprost sodium, which had been initiated 1 month prior and escalated to 120 μg daily 1 week before admission. On hospital day (HD) 3, he underwent thromboendarterectomy. After an uneventful overnight stay in the intensive care unit (ICU), he was transferred to a general ward. His initial postoperative course was stable until HD 7, when he developed acute hypoxemic respiratory failure (PaO_2_/FiO_2_ ratio [P/F ratio], 150 mmHg) following an episode of vomiting. He was then emergently readmitted to the ICU and intubated. Initial investigations, including chest CT on HD 7, revealed newly developed bilateral infiltrates, pleural effusion, and atelectasis (**Fig. 1A**). Under mechanical ventilation with a positive end-expiratory pressure of 7 cmH_2_O, his P/F ratio dropped to the 100s. Transthoracic echocardiography ruled out cardiogenic pulmonary edema and fluid overload. Based on these findings, we made a working diagnosis of suspected acute respiratory distress syndrome complicated by aspiration pneumonitis, pleural effusion, and atelectasis.

Broad-spectrum antibiotics and several days of prone positioning therapy were implemented. After intubation, beraprost sodium was administered via a nasogastric tube. Although decreased gastrointestinal peristalsis is typically a concern after vomiting-induced respiratory failure, abdominal evaluation via CT showed no findings suggestive of bowel obstruction, and the nasogastric aspirate volume was minimal, indicating adequate enteral tolerance. Furthermore, to maintain peripheral blood flow for his critical limb ischemia, we resumed medication early without interruption. These interventions led to an initial improvement in oxygenation, with the P/F ratio increasing to approximately 200 mmHg. Following successful spontaneous awakening trials and spontaneous breathing trials (SBTs), extubation was attempted on HD 10 with no success. Subsequently, his respiratory recovery was protracted, with the P/F ratio fluctuating between 150 and 250 mmHg. Given the persistent oxygenation impairment and copious airway secretions, weaning was deemed difficult. Consequently, tracheostomy was performed on HD 25 for long-term airway management.

Throughout his ICU stay, intermittent episodes of facial flushing, tachypnea, tachycardia, and hypertension were observed multiple times daily. These signs were initially attributed to patient–ventilator asynchrony or a physiological stress response to discomfort. Although ventilator settings were optimized and sedative regimens adjusted, these interventions provided no sustained relief, prompting further investigation. Concurrently, a follow-up CT scan on HD 29 demonstrated a significant interval increase in these effusions, resulting in extensive compressive atelectasis, particularly in the lower lobes (**Fig. 1B**). These were managed with diuretics and a single therapeutic thoracentesis. Although thoracentesis yielded transient improvement in gas exchange, the paroxysmal desaturation events persisted, deepening the diagnostic enigma. In our department, we strictly adhere to the joint protocol for weaning from mechanical ventilation issued by 3 major Japanese societies.^5)^ Because this protocol defines a continuous SpO_2_ level of >94% as a criterion for successful SBT, any drop in SpO_2_ below 94% during the trial was consistently treated as an SBT failure in this case.

Post-ICU discharge, weaning attempts were consistently hindered by the recurrence of tachypnea, desaturation, tachycardia, and facial flushing during SBTs. On HD 44, these paroxysmal episodes occurred independently of weaning trials. Arterial blood gas analysis during an event revealed acute respiratory alkalosis with hypoxemia. A potential link to intermittent enteral feeding was initially considered and empirically addressed by switching to continuous infusion. Concurrently, considering that the constellation of symptoms, particularly facial flushing, resembled the known vasodilatory adverse effects of beraprost sodium, a drug-induced etiology was suspected. Consequently, beraprost sodium was discontinued on HD 49.

Cardiorespiratory instability resolved the next day (HD 50). The patient subsequently tolerated a return to intermittent enteral feeding and achieved prolonged periods of daytime liberation from the ventilator. He was fully liberated from mechanical ventilation on HD 53. After successful liberation from mechanical ventilation, the patient was weaned off high-flow tracheal oxygenation and remained stable on room air. Given that the initial indication for beraprost sodium was to manage the critical limb ischemia, and in light of the improvement in his overall condition, including respiratory status, a decision was made to cautiously reintroduce the medication on HD 66 with close cardiorespiratory monitoring. Approximately 45 min after administering a single 40 μg dose, he experienced an acute recurrence of facial flushing, tachypnea (30 breaths/min), and desaturation (SpO_2_ 88% on room air), necessitating 3 L/min of supplemental oxygen. The prompt recurrence of this distinct symptom complex upon re-exposure strongly implicated beraprost sodium as the causative agent. Therefore, the drug was immediately and permanently discontinued. His respiratory status returned to baseline within 5 h, and supplemental oxygen was no longer required.

**Fig. 2:**
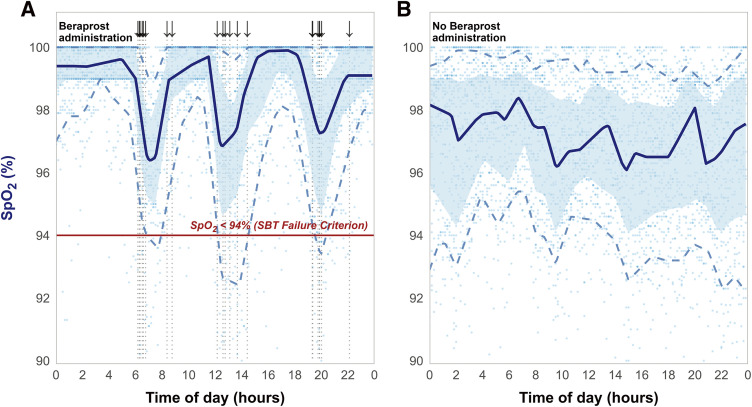
A comparison of composite 24-h SpO_2_ profiles during beraprost administration with those obtained after drug discontinuation during ventilator weaning. To visualize the daily recurrence of desaturation events, data from 28 consecutive days in each period were superimposed onto a single 24-h x-axis. Continuous SpO_2_ data (automatically recorded at 1-min intervals) were extracted from the electronic medical record and aggregated into 10-min averages using R software (version 4.3.3; R Foundation for Statistical Computing, Vienna, Austria) to reduce short-term noise and visually clarify diurnal trends. Light blue dots represent these averaged individual SpO_2_ measurements from all days within the specified period. The solid dark blue line represents the median (50th percentile), the shaded area represents the interquartile range (25th–75th percentile), and the dashed lines represent the 10th and 90th percentiles. (**A**) During beraprost administration and mechanical ventilation (hospital days 22–49), recurrent, severe, and nonphysiological desaturation episodes were observed. These episodes were frequently temporally associated with beraprost administration (arrows and vertical dotted lines) and often fell below the SBT failure threshold of 94% (red line). The downward arrows indicate the exact beraprost administration times extracted from the daily nursing records. Because data from multiple days are superimposed, these arrows cluster around the scheduled administration time blocks. (**B**) After discontinuation of beraprost (dechallenge; hospital days 50–77), cyclical desaturation events were absent, and SpO_2_ remained stable throughout the 24-h cycle. SBT: spontaneous breathing trial

To retrospectively evaluate the relationship between recurrent desaturation episodes and medication, continuous SpO_2_ monitoring data (recorded at 1-min intervals) and precise beraprost administration times were extracted from electronic medical and nursing records. To minimize short-term noise and clarify diurnal trends, the 1-min SpO_2_ data were aggregated into 10-min intervals. The average value for each segment was then plotted using R software (version 4.3.3) (**Fig. 2**).

## Discussion

The clinical course of this patient, specifically the resolution of respiratory deterioration upon dechallenge and its recurrence upon rechallenge, suggests that oral beraprost may have contributed to the prolonged ventilator weaning failure. Furthermore, these clinical observations indicate that this potential adverse effect may be reversible upon drug discontinuation. Rather than representing an idiosyncratic drug reaction, this finding highlights a predictable, context-dependent adverse effect resulting from a patient–drug interaction in a uniquely susceptible individual.

Although the patient had taken beraprost sodium for approximately 1 month before surgery without any respiratory symptoms, respiratory failure occurred postoperatively. This temporal pattern can be explained by postoperative pathophysiological changes. Postoperatively, the patient developed extensive atelectasis and pleural effusion, with HPV being critical in maintaining oxygenation. In this context, the inhibitory effect of beraprost on HPV likely became clinically significant. Furthermore, several postoperative factors, such as systemic inflammation, decreased plasma albumin levels, altered pulmonary perfusion, and increased free fraction of the drug, may have enhanced the pulmonary exposure and vasodilatory effect of beraprost, even at a relatively low dose. Collectively, these mechanisms provide a plausible explanation for the delayed onset and heightened sensitivity observed postoperatively.

The immediate mechanism of the weaning failure was a distinct clinical syndrome characterized by (1) impaired gas exchange (hypoxemia) and (2) an unsustainable breathing pattern (tachypnea with preserved tidal volume). The hypoxemia is best explained by HPV inhibition in the setting of extensive atelectasis (**Fig. 1B**), leading to worsened V/Q mismatch and intrapulmonary shunting.^3)^ This case exemplifies the long-standing principle that systemic vasodilators can unmask clinically significant shunts in patients with underlying lung disease, a concept well recognized in conditions such as chronic obstructive pulmonary disease-associated pulmonary hypertension.^6)^ The recurrent desaturation episodes (**Fig. 2A**), which was resolved after drug cessation (**Fig. 2B**), are clinically consistent with this potential mechanism.

**Fig. 3:**
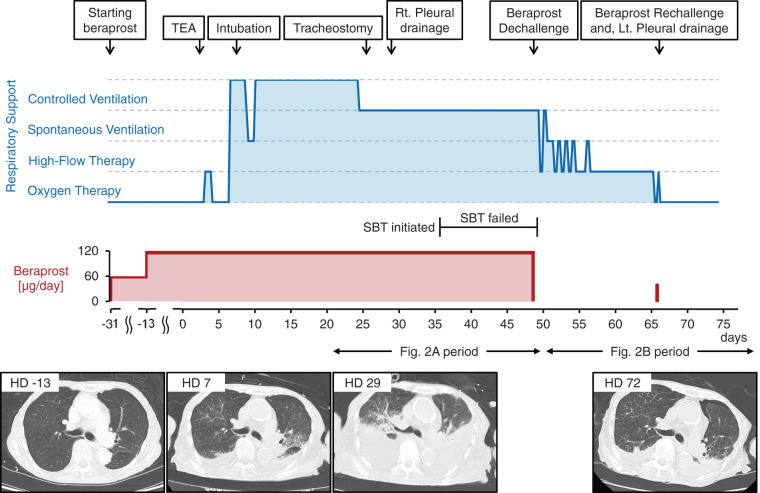
Timeline illustrating the patient’s clinical course. The upper section shows the major clinical events, level of respiratory support (blue graph), and dosage of beraprost sodium (red graph) over time, while the lower section displays serial chest CT images. Horizontal bidirectional arrows denote the “**Fig. 2A** period” (hospital days 22–49) and the “**Fig. 2B** period” (hospital days 50–77), linking the clinical timeline to the composite SpO_2_ data. After discontinuation of beraprost on hospital day 49, the level of respiratory support was gradually de-escalated. Coinciding with a single-dose rechallenge on hospital day 66, respiratory support was transiently increased. HD: hospital day; SBT: spontaneous breathing trial; TEA: thromboendarterectomy; Rt.: right; Lt.: left

The slightly lower and more variable baseline SpO_2_ in **Fig. 2B** reflects the expected clinical transition from stable mechanical ventilation to high-flow nasal cannulation or traditional oxygen therapy following successful liberation from the ventilator. This anticipated variability was influenced by the cessation of positive pressure ventilation, reduced target SpO_2_ for post-extubation management (>94%), and frequent FiO_2_ titration within the prescribed range by nurses as the patient improved. Most importantly, the sudden, atypical, and precipitous SpO_2_ drops observed during beraprost administration completely disappeared after drug discontinuation. In parallel, the marked tachypnea with preserved tidal volume suggests a potential stimulation of the respiratory drive beyond simple compensation for hypoxemia. Although formal physiological assessments were not performed in this case, which limited our ability to determine the exact underlying mechanisms, we hypothesized, based on our clinical observations and the known pharmacological profiles of prostacyclin analogues,^7,8)^ that these findings might reflect a vicious cycle of gas exchange impairment and heightened respiratory drive, which complicated the weaning process. The temporal association between beraprost administration and this complex respiratory failure is clearly visualized in **Figs. 2** and **3**.

The rarity of previous reports may reflect trial exclusion criteria in landmark prostacyclin studies.^2)^ Nonetheless, this case aligns with clinical scenarios such as acute pulmonary embolism, where dyspnea often exceeds the degree of hypoxemia, implicating mechanoreceptor stimulation. The route of administration is crucial; inhaled prostacyclins minimize systemic hemodynamic effects and V/Q mismatch,^9,10)^ underscoring the need for careful drug selection.

Despite this adverse event, we do not intend to discourage the clinical use of beraprost. Following this experience, our institution has neither discontinued nor avoided beraprost use in other patients when clinically indicated. Rather, this case has provided an important lesson to help recognize and prevent potential patient–drug interactions in similar postoperative settings in the future.

## Conclusion

Oral beraprost can precipitate severe recurrent ventilator weaning failure in patients with underlying V/Q mismatch. Awareness of this potential adverse effect is critical to avoid unnecessary prolonged mechanical ventilation and its associated complications. When an unexplained weaning failure is encountered, a comprehensive review of medications, including systemic vasodilators, is essential. We recommend that a wide range of medical professionals, including vascular surgeons, intensivists, and pharmacists involved in ward and ICU medication management, remain vigilant regarding this potential patient–drug interaction.

## Declarations

### Data availability

The data supporting the findings of this study are available from the corresponding author upon reasonable request. The data are not publicly available due to privacy and ethical restrictions.
